# Fexinidazole interferes with the growth and structural organization of *Trypanosoma cruzi*

**DOI:** 10.1038/s41598-022-23941-z

**Published:** 2022-11-27

**Authors:** Aline Araujo Zuma, Wanderley de Souza

**Affiliations:** 1 Laboratorio de Ultraestrutura Celular Hertha Meyer, Instituto de Biofísica Carlos Chagas Filho, Universidade Federal Do Rio de Janeiro, Av. Carlos Chagas Filho, 373, Centro de Ciências da Saúde, Cidade Universitária, Ilha do Fundão, Rio de Janeiro, RJ 21491-590 Brazil; 2 Centro Multidisciplinar de Pesquisas Biológica-CMABio, Escola Superior de Ciências da Saúde, Universidade do Estado do Amazonas-UEA, Av. Carvalho Leal, 1777-Cachoeirinha, Manaus, AM 69065-000 Brazil

**Keywords:** Target identification, Parasitic infection

## Abstract

Fexinidazole (FEX) is a heterocyclic compound and constitutes the first 100% oral treatment drug for African trypanosomiasis. Its effectiveness against *Trypanosoma brucei* encouraged the investigation of its antiparasitic potential against *T. cruzi*, the aetiological agent of Chagas disease. Although previous studies addressed the antitrypanosomal effects of FEX, none used electron microscopy to identify the main target structures of *T. brucei* or *T. cruzi. In* this work, we used microscopy techniques to analyze the ultrastructural alterations caused by FEX in different developmental stages of *T. cruzi*. In addition to inhibiting *T. cruzi* proliferation, with IC_50_ of 1 µM for intracellular amastigotes, FEX promoted massive disorganization of reservosomes, the detachment of the plasma membrane, unpacking of nuclear heterochromatin, mitochondrial swelling, Golgi disruption and alterations in the kinetoplast-mitochondrion complex. Together, these observations point to FEX as a potential drug leader for further developing of chemotherapy against Chagas disease.

## Introduction

Azoles are a class of heterocyclic compounds widely used to treat fungal infections. They act on inhibiting of the fungal cytochrome P450 (P450) from family 51 (CYP51 or sterol 14a-demethylase), indispensable for ergosterol biosynthesis^1^. Fexinidazole (FEX), a 2-substituted 5-nitroimidazole, is a prodrug whose activity depends on two-electron reductions of the NO_2_ group by an NADH-specific nitroreductase (TbNTR1) in *Trypanosoma brucei*. *T. cruzi* also presents an orthologous nitroreductase, which acts on the activation of nitroheterocycle compounds, such as benznidazole and nifurtimox. The amine species generated by FEX are toxic and mutagenic to trypanosomes^2^.

The antiparasitic potential of FEX began to be investigated by Hoechst between the 1970s and 1980s. However, these studies did not progress to the clinical stage until years later, when DND*i* selected FEX, from more than 700 nitroheterocyclic compounds, as a new drug candidate for treating African trypanosomiasis (HAT)^3,4^. This disease occurs in two forms: (1) *T. brucei gambiense* causes gambiense HAT in West and Central Africa, is characterized by the chronic form and can take several years between infection and death. (2) *T. brucei rhodesiense* is the causative agent of rhodesiense HAT in East and Southern Africa. In this case, the interval between infection and the patient's death may be only a few weeks or months^5,6^.

Aiming to include FEX in clinical trials, its pharmacological and toxicological properties have been extensively studied. Since the results indicated that FEX had good tolerance, between 2010 and 2011, DND*i* (Drugs for Neglected Diseases initiative) started the safety and pharmacokinetic evaluation in human volunteers by administering single and multiple doses^3,4^. As a result, FEX became the first 100% oral treatment drug for the first and second stages of HAT, further reducing the need for hospitalization. Phase 3 is in progress in the Democratic Republic of Congo, which has the most HAT cases in Africa and Guinea^2^.

The promising results of FEX against *T. brucei* led different research groups to investigate its antiparasitic potential against *T. cruzi*, the aetiological agent of Chagas disease, which is a Neglected Tropical Disease (NTD). The treatment of Chagas disease is based on benznidazole and nifurtimox the only available drugs, although they are not effective during the chronic phase and cause several side effects^7^.

FEX was shown to be active against *T. cruzi* Brazil 32 strain trypomastigotes reducing the number of circulating parasites in the blood of infected mice^8^. The activity of FEX against different *T. cruzi* strains (CL Brener, Y, Colombian, and VL-10) have been evaluated in vivo and was compared to the reference drug benznidazole. FEX suppressed parasitemia, prevented animal death for all strains, and reduced myocarditis in 100% of animals infected with VL-10 or Colombian strains^4^. In 2016, Francisco et al. showed that FEX and fexinidazole sulfone were more effective than benznidazole against *T. cruzi* CL Brener strain infected animals^9^. In 2017, Spain started a new phase II proof-of-concept study using shorter, lower-dose treatment regimens for Chagas disease. This study suggests that treatment of up to 10 days is effective. However, the minimum dose required and the risk–benefit ratio need further investigation^10^.

Although several reports in the literature address the in vivo antitrypanosomal effects of FEX, including its mechanism of action and clinical trials, no studies describe the ultrastructural changes caused by this drug on *T. brucei* or *T. cruzi*. Previous studies have shown that electron microscopy analysis of drug-treated parasites may contribute to the identification of parasite target structures and organelles. Our present observation indicates that this compound inhibits parasite proliferation (both in axenic epimastigotes and intracellular amastigotes) and induces significant lesions in structures such as the plasma membrane, the reservosomes, and the Golgi complex, and unpacking of the nuclear heterochromatin. It also interferes with cell cycle progression, decreasing the percentage of cells at the G1 and increasing at G2/M.

## Results

### Parasite proliferation and viability

**Figure 1:**
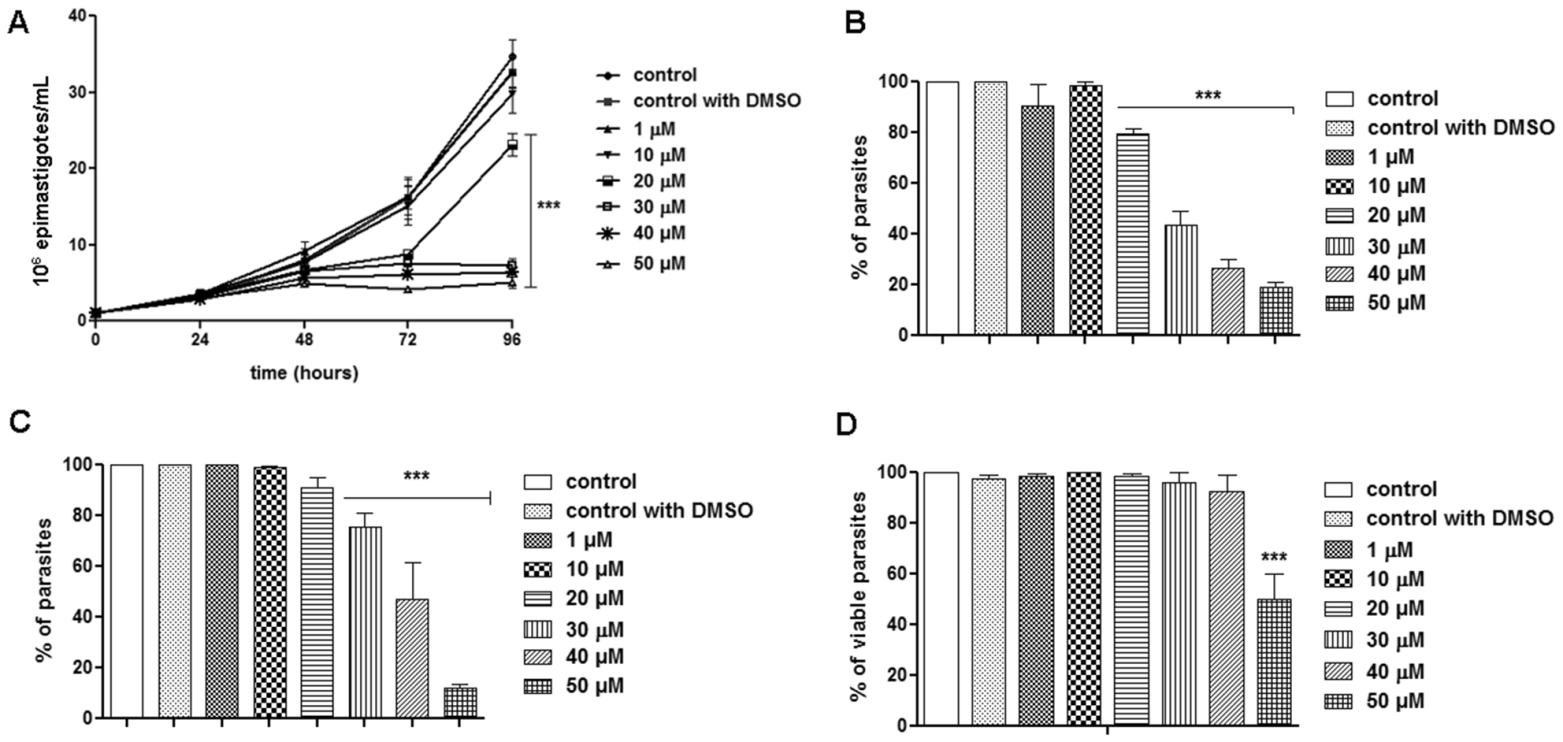
*T. cruzi* epimastigotes proliferation and viability in the presence of FEX. (**A**) Epimastigote proliferation was strongly inhibited for up to 72 h of treatment. (**B**) The percentage of parasites after 72 h in the presence of the drug (i.e., 96 h of growth) indicates that the most effective concentrations were 30, 40, and 50 µM. (**C**) After removal of the drug from the medium, the percentage of parasites indicates an increase in the number of epimastigotes. (**D**) Epimastigote viability after 72 h of treatment. The percentage of viable parasites was similar, except in the presence of 50 µM. The data are the average of three independent experiments in triplicate (***p < 0.001).

FEX was effective in inhibiting epimastigote proliferation of the *T. cruzi* Y strain. Its effect was time-dependent but at the highest concentrations (30, 40, and 50 µM), the number of parasites remained similar, suggesting interference in cell cycle progression (Fig. 1A). After 72 h of treatment (i.e., 96 h of growth), the percentage of parasites treated with 20, 30, 40, and 50 µM was 80, 43, 26, and 19%, respectively, relative to the control group (Fig. 1B). The IC_50_ values were 40 ± 10, 30 ± 10, and 23 ± 3 µM after 24, 48, and 72 h of treatment, respectively (Table 1). Interestingly, despite the strong inhibition caused up to 72 h of treatment, after removing the drug from the culture medium, the antiproliferative effect of FEX was reversible (Fig. 1C). Under this condition, the number of parasites increased, and those previously treated with 20 µM represented 91% compared to the control, those treated with 30 µM reached 75% and the epimastigotes treated with 40 µM represented 47% compared to non-treated parasites. The only exception was the group treated with the highest dose (50 µM), whose percentage of parasites compared to the control was 12%. Epimastigote viability in the presence of FEX for 72 h was also evaluated. There was no reduction in the number of viable parasites, except at the concentration of 50 µM. In this case, the percentage was 50% lower than untreated epimastigotes (Fig. 1D).

**Table 1:** Analysis of cytotoxicity and trypanocidal effect of FEX.

IC_50_ (µM) *T. cruzi* Epimastigote			IC_50_ (µM) *T. cruzi* Amastigote	SI	LD_50_ (µM) *T. cruzi* Trypomastigote	SI	CC_50_ (µM) LLC-MK_2_
*24 h*	48 h	72 h	72 h		24 h		96 h
40 ± 10 µM	30 ± 10 µM	23 ± 3 µM	1 ± 0.5 µM	80	50 ± 2 µM	1.6	80 ± 10 µM

IC_50_: concentration that inhibits 50% of the proliferation of the replicative form.

LD_50_: concentration that causes lysis in 50% of the trypomastigotes.

CC_50_: concentration that inhibits the proliferation of host cells by 50%.

SI: selectivity index.

**Figure 2:**
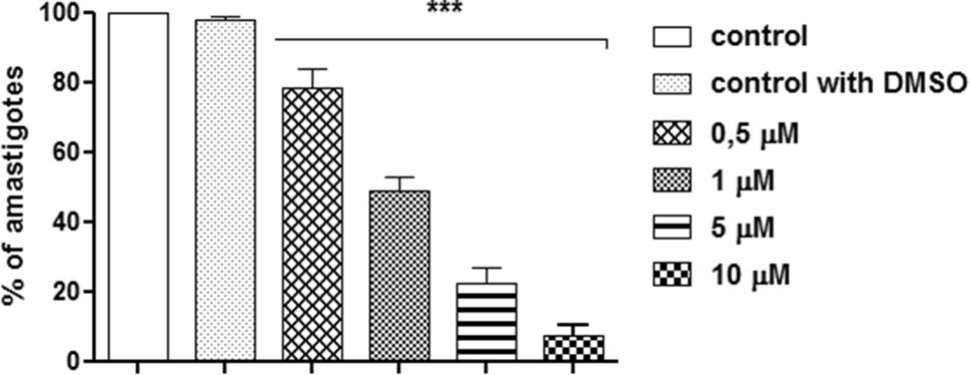
*T. cruzi* amastigotes proliferation in the presence of FEX. The number of parasites was reduced in a dose-dependent way. The number of parasites reduced from 20% after treatment with 0.5 µM to 93% with 10 µM. The data are the average of three independent experiments in triplicate (***p < 0.001).

The trypanocidal effect of FEX was investigated against intracellular amastigotes after 72 h of treatment. FEX reduced amastigote proliferation dose-dependently (Fig. 2). In the presence of 0.5 µM, the number of amastigotes corresponded to 80% of the control group. After treatment with 1, 5, and 10 µM, the percentage of parasites was equivalent to 50, 22, and 7%, respectively, compared to the untreated group. The IC_50_ value of FEX against *T. cruzi* amastigotes was 1 ± 0.5 µM µM (Table 1).

**Figure 3:**
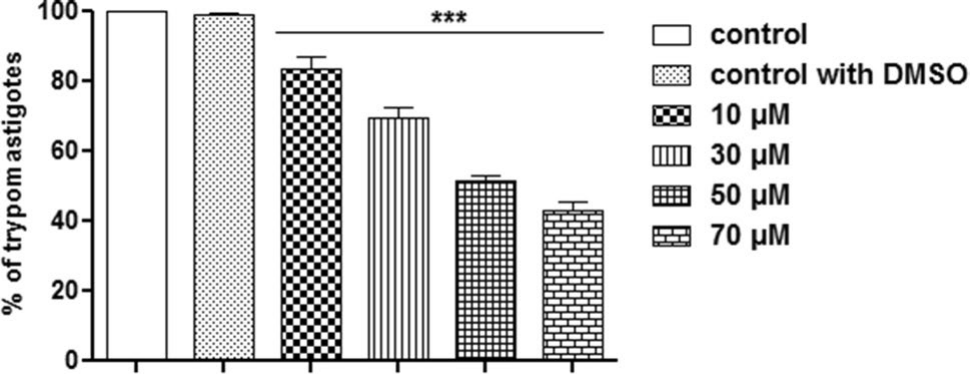
Trypanocidal effect of FEX against trypomastigotes after 24 h. The number of parasites was reduced in a dose-dependent manner. The data are the average of three independent experiments in triplicate (***p < 0.001).

In addition to being effective against epimastigotes and amastigotes, FEX caused a reduction in the number of trypomastigotes in a dose-dependent manner (Fig. 3). After 24 h of treatment, in the presence of 10 μM, there were almost 20% fewer parasites. However, this percentage increased to more than 50% fewer parasites with 70 µM. With this data, the LD_50_ of FEX was 50 µM ± 2 µM (Table 1).

### *T. cruzi* ultrastructure

To investigate the effects of FEX on *T. cruzi* ultrastructure, epimastigotes treated with 20, 30, and 40 µM for 72 h were fixed and prepared as described in Sect. “Transmission electron microscopy”. Under these conditions, the inhibition of proliferation was more apparent while the parasite remained viable.

**Figure 4:**
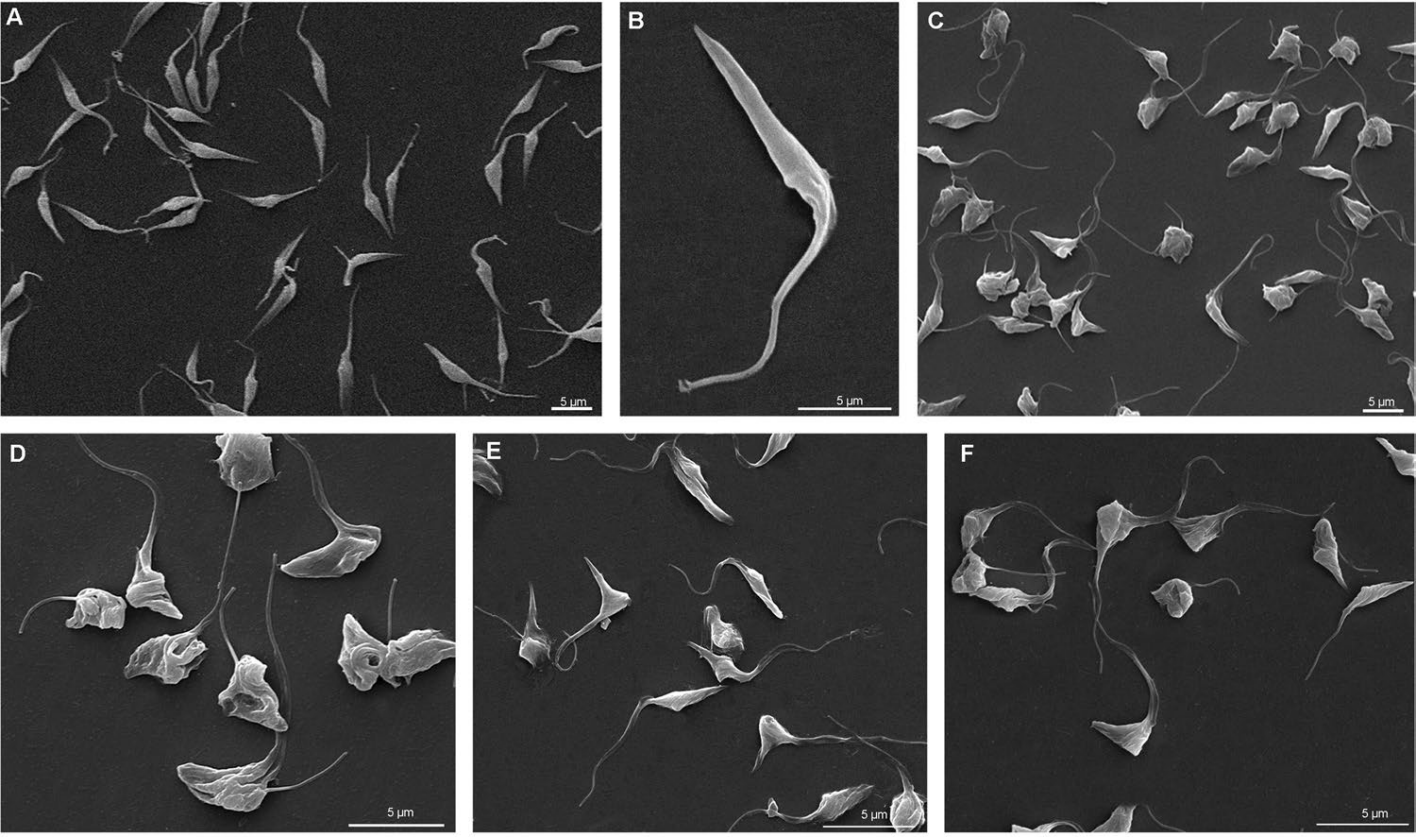
Scanning electron microscopy of *T. cruzi* epimastigotes in the presence of FEX for 72 h. (**A**, **B**) Control epimastigotes showing the typical elongated shape. (**C**, **D**) Treatment with 20 µM. (**E**) Treatment with 30 µM. (**F**) Treatment with 40 µM. FEX caused rounding and wrinkling of parasites.

Scanning electron microscopy was used to evaluate *T. cruzi* morphology in the presence of FEX. Control epimastigotes display a typical elongated shape (Fig. 4A,B) that was strongly affected after treatment with 20 µM (Fig. 4C,D), 30 µM (Fig. 4E), and 40 µM (Fig. 4F). Although parasites were mostly rounded and wrinkled, the frequency or intensity of these modifications was not dose-dependent.

**Figure 5:**
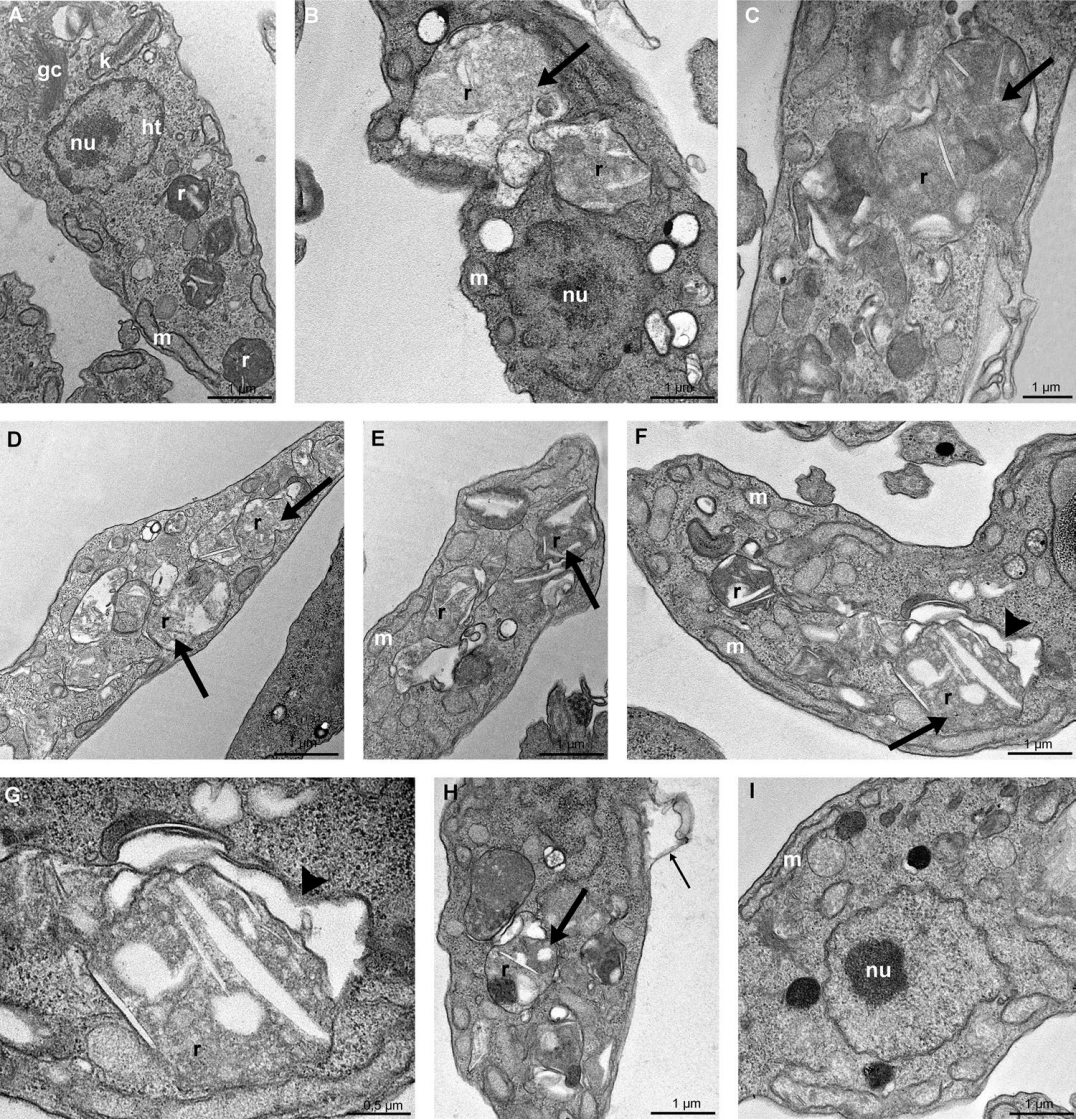
Transmission electron microscopy of *T. cruzi* epimastigotes in the presence of FEX for 72 h. (**A**) Non-treated epimastigote with the Golgi complex (GC) near the bar-shaped kinetoplast (k), the nucleus with the condensed heterochromatin (ht) close to the nuclear envelope and around the nucleolus (nu), a single branched mitochondrion (m) and reservosomes (r) at the posterior end. (**B**–**E**) Treatment with 20 µM (**B**, **C**) and 30 µM (**D**, **E**) caused a loss of rounded shape and matrix content of the reservosomes (thick arrow). (**F**–**I**) Treatment with 40 µM also caused the detachment of the reservosomes from the cytoplasm (**F**, **G**, arrowhead), detachment of the plasma membrane (**H**, thin arrow), and the unpacking of nuclear heterochromatin (**I**).

Non-treated epimastigotes are elongated parasites with the Golgi complex near the bar-shaped kinetoplast, the nucleus with the condensed heterochromatin close to the nuclear envelope, and around the nucleolus, a single branched mitochondrion and reservosomes at the posterior end (Fig. 5A), as revealed by transmission electron microscopy. In general, FEX promoted massive disorganization of reservosomes. Treatment with 20 µM (Fig. 5B,C) and 30 µM (Fig. 5D,E) caused a loss of the rounded shape and matrix content of the reservosomes. On treated parasites, it is also remarkable the presence of many lipid inclusions. In the presence of 40 µM, FEX also led to cytosolic extraction near the reservosomes (Fig. 5F,G). Moreover, the detachment of the plasma membrane and the unpacking of nuclear heterochromatin were reported (Fig. 5H,I). It is worth mentioning that under these circumstances, FEX did not cause other common drug-induced ultrastructural alterations, such as mitochondrial swelling, Golgi disruption, or loss of kDNA topology.

**Figure 6:**
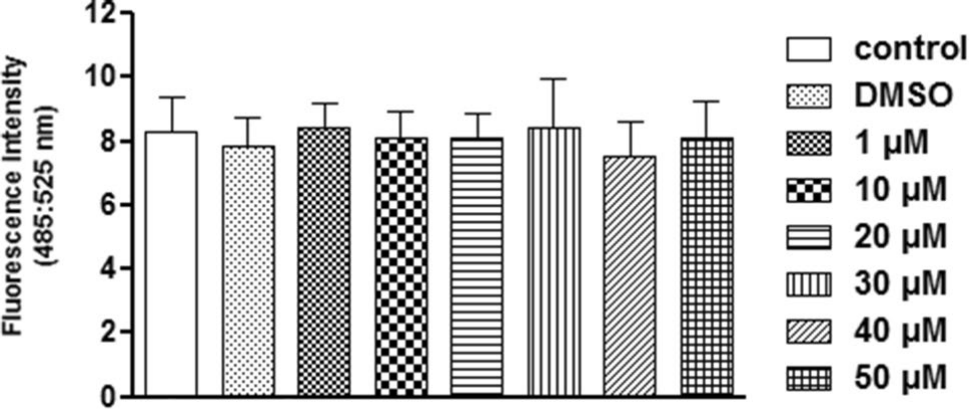
Neutral lipid quantification on *T. cruzi* epimastigotes in the presence of FEX for 72 h. The number of neutral lipids in treated and untreated parasites was similar. The data are the average of three independent experiments in triplicate.

Considering the changes reported in the reservosomes, we analyzed whether the 72-h treatment with FEX would change the number of neutral lipids on *T. cruzi* epimastigotes. Nile red staining showed that none of the concentrations tested caused a significant difference in the quantification of neutral lipids compared to the control group (Fig. 6).

**Figure 7:**
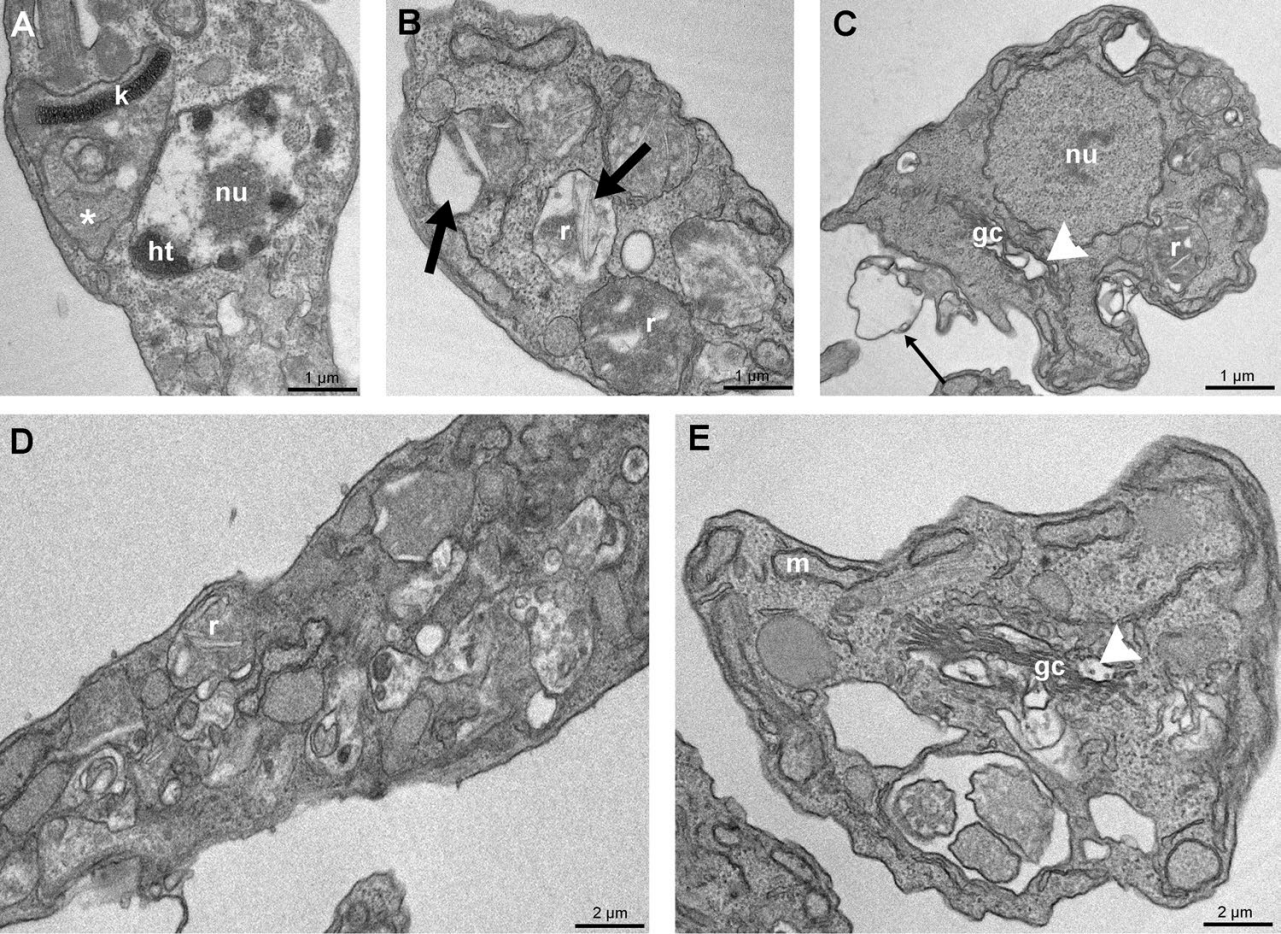
Transmission electron microscopy of *T. cruzi* epimastigotes after removing FEX from the medium. (**A**) Parasites previously treated with 20 µM presented kinetoplast swelling (asterisk). (**B**) Epimastigotes treated with 30 µM showed alterations on reservosomes ultrastructure (thick arrow). (**C**–**E**) Parasites were treated with 40 µM. Note the unpacking of nuclear heterochromatin, plasma membrane detachment (thin arrow), intense cytoplasmic disorganization contrary to the continuous treatment, and Golgi complex disruption (white arrowhead).

The effects described above were observed during 72 h of treatment with FEX. Therefore, we further investigated whether there would be some ultrastructural reorganization of the previously treated parasites after removing the drug from the medium. After 72 h in the absence of the drug, mitochondrial swelling at the kinetoplast region was observed on epimastigotes that were previously treated with 20 µM (Fig. 7A). Reservosomes modification was still present, as shown in Fig. 7B,D, regardless of the concentration that was initially used. Epimastigotes previously treated with 40 µM also presented unpacking nuclear heterochromatin, plasma membrane detachment (Fig. 7C) and Golgi complex disorganization (Fig. 7C,E), the latter unlike what was reported in continuous treatment.

Scanning electron microscopy also analyzed the reversibility in the ultrastructure of epimastigotes. However, in this case, there were no changes since the morphology of the parasites remained the same (data not shown) compared to Fig. 5.

**Figure 8:**
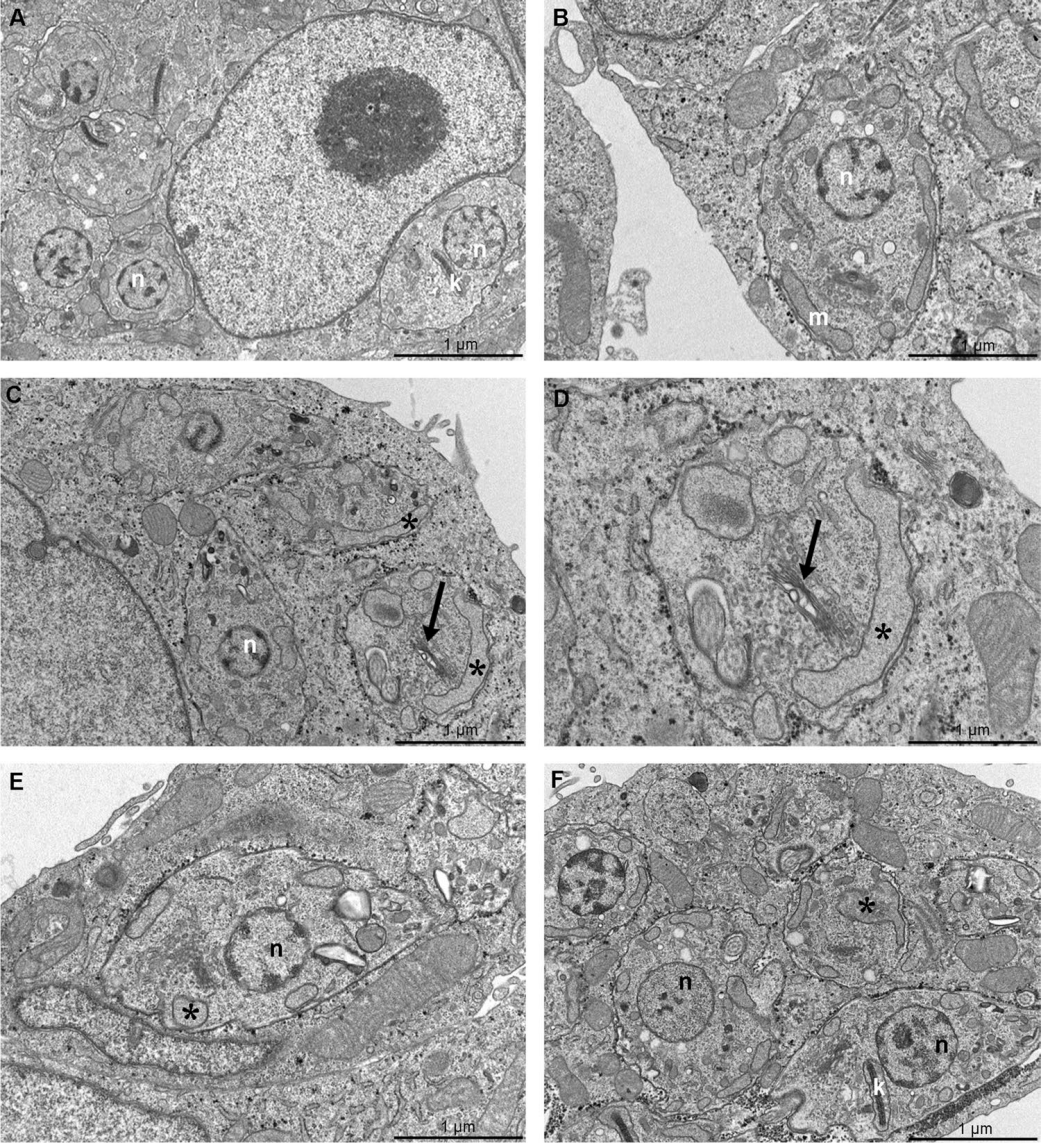
Transmission electron microscopy of *T. cruzi* amastigotes in the presence of FEX for 72 h. (**A**) Non-treated amastigotes present the nucleus (n) with the condensed heterochromatin, a single branched mitochondrion (m), and a bar-shaped kinetoplast (k). (**B**) Five-hundred nanomolar did not promote ultrastructural modifications on amastigotes. (**C**, **D**) One micromolar caused Golgi disorganization (black arrow) and mitochondrial swelling (asterisk). (**E**, **F**) Amastigotes treated with 5 µM presented mitochondrial swelling (asterisk).

The effects of FEX on amastigotes ultrastructure were also investigated in the presence of 0.5, 1, and 5 µM after 72 h. Non-treated amastigotes have an organization similar to that of epimastigotes (Fig. 8A). At the lowest concentration of FEX, no alterations were observed in the parasites (Fig. 8B). In the presence of 1 µM, amastigotes presented disorganization of Golgi complex and mitochondrial swelling (Fig. 8C,D), which was also found after treatment with 5 µM (Fig. 8E,F). It is worth mentioning that host cells did not show ultrastructural modifications in the presence of the drug.

**Figure 9:**
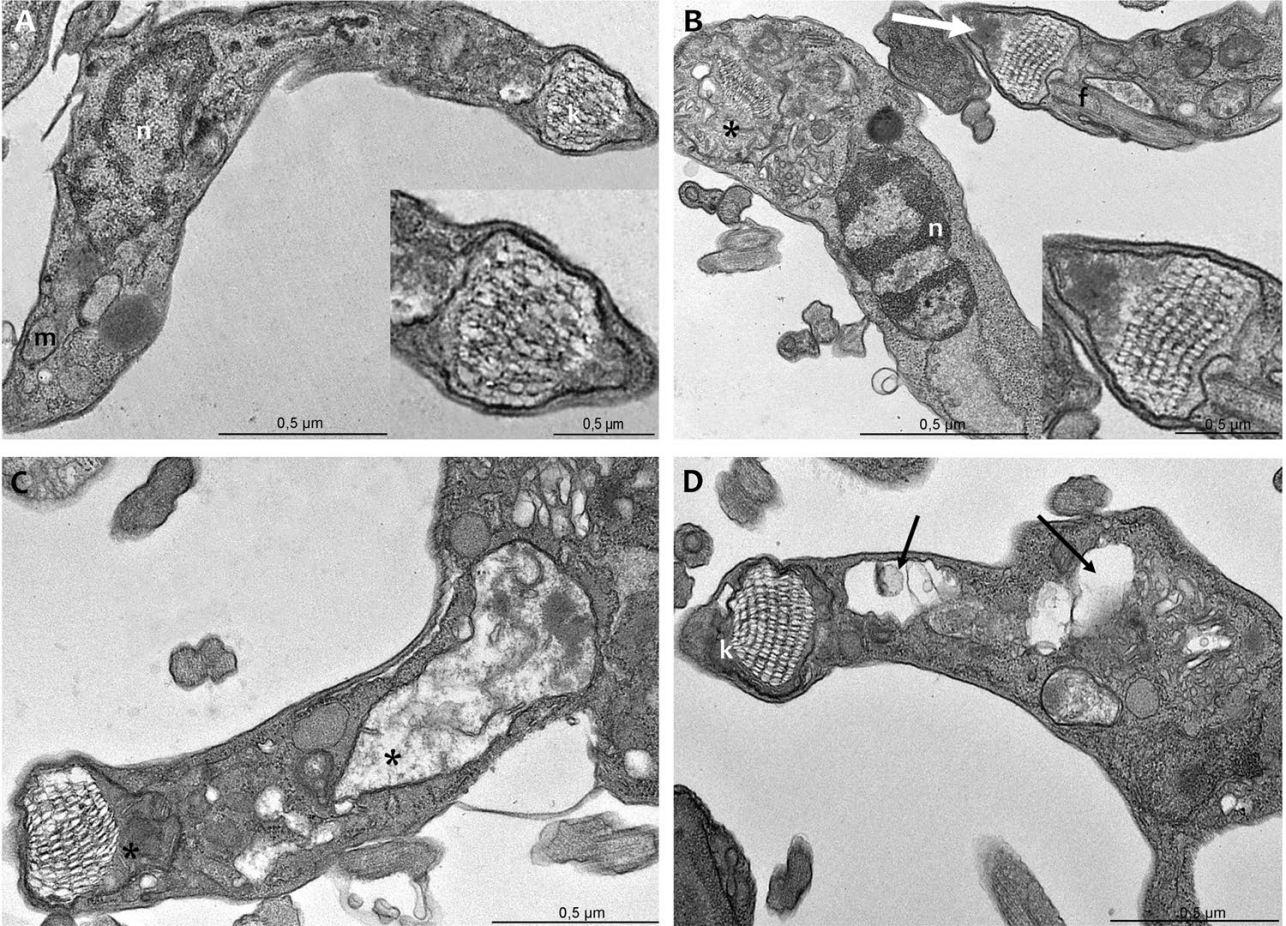
Transmission electron microscopy of *T. cruzi* trypomastigotes in the presence of FEX for 24 h. (**A**) Non-treated parasites present an elongated shape, an oval nucleus (n), a single branched mitochondrion (m), and a more relaxed basket-shaped kDNA (k). (**B**) Ten micromolar caused mitochondrial swelling (asterisk) and the appearance of unusual electron-dense content between the kDNA and the mitochondrial membrane (white arrow). (**C**) With 30 µM, parasites presented mitochondria swelling (asterisk). (**D**) Treatment with 50 µM caused vacuolization of cytoplasm (black arrow).

FEX-treated trypomastigotes were analyzed by transmission electron microscopy, which revealed alterations in mitochondria and more specifically in the kinetoplast. Non-treated trypomastigotes are elongated parasites with an oval nucleus, a single branched mitochondrion, and a more relaxed basket-shape kDNA (Fig. 9A). After treatment with 10 µM for 24 h, parasites presented mitochondrial swelling and some unusual electron-dense content between the kDNA and the mitochondrial membrane (Fig. 9B). However, the kDNA topology was similar to that of the control parasites. In the presence of 30 µM, the mitochondria were still affected (Fig. 9C), and with 50 µM, cytoplasmic vacuoles were also found in trypomastigotes (Fig. 9D).

### *T. cruzi* cell cycle

**Figure 10:**
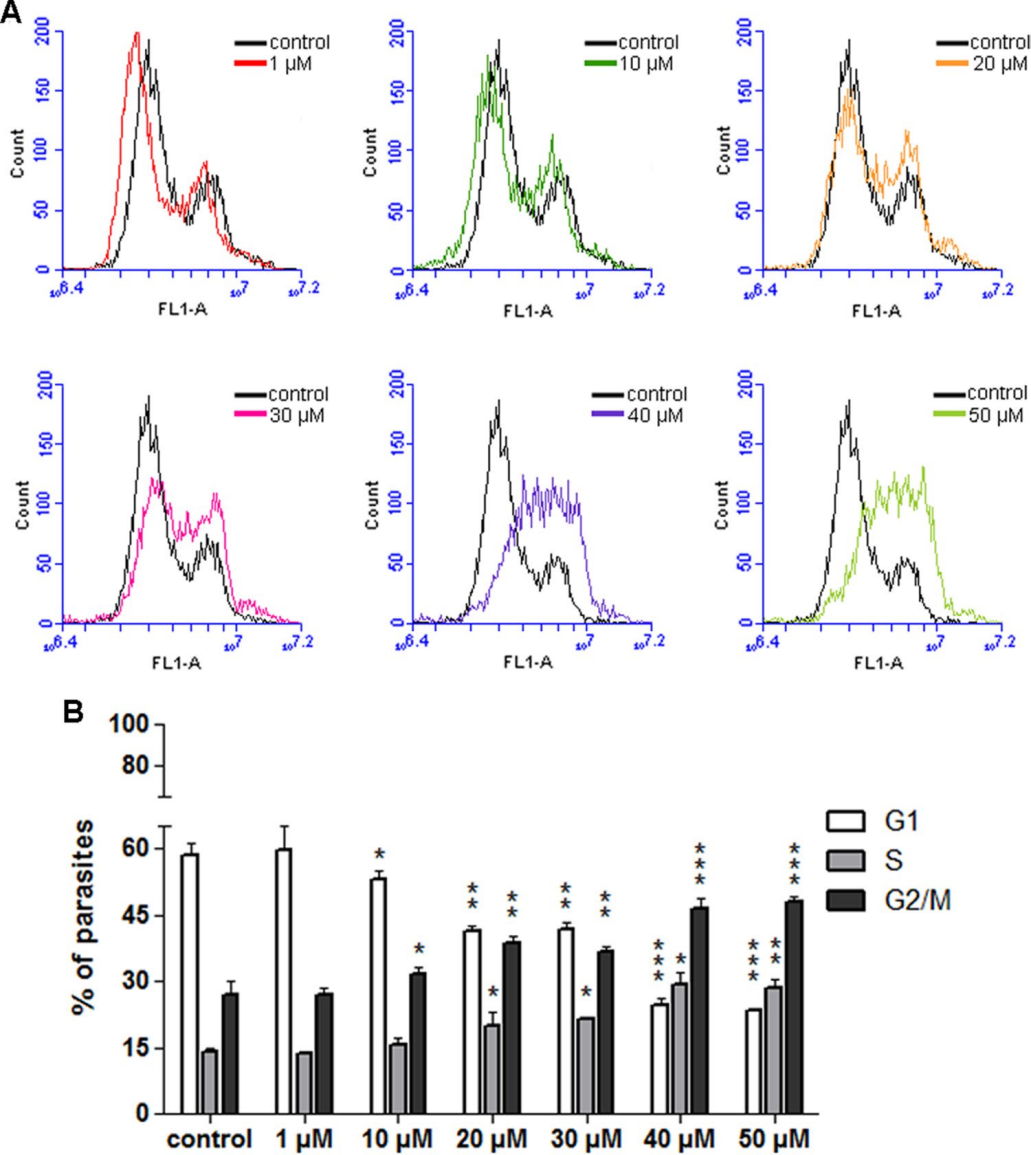
Epimastigotes cell cycle in the presence of FEX for 72 h. (**A**) Histograms of control and treated parasites. Black and color lines indicate untreated and treated epimastigotes, respectively. (**B**) Percentage of parasites in G1, S, and G2/M. In the control group, most epimastigotes were in the G1 phase, followed by S and G2/M. From 10 µM, there was a lower number of parasites in G1 and a higher percentage in G2/M. This pattern became more pronounced at higher concentrations. The histograms are representative and the data are the average of two independent experiments (*p > 0.05; **p < 0.05; ***p < 0.001).

The data regarding epimastigotes proliferation and ultrastructure (shown in Figs. 1A and 5, respectively) indicated a possible cell cycle blockade in the presence of 30, 40, and 50 µM FEX. Based on that, we performed a cell cycle analysis after 72 h of treatment. As expected, in the control group, most epimastigotes were in the G1 phase (58.6%), followed by 14.4% in S and 27% in G2/M (Fig. 10A,B). The data after treatment with 1 µM were similar to those of non-treated parasites (59.7; 13.5 and 26.8% in G1, S, and G2/M, respectively). From 10 µM, there was a slight difference (53, 15.5 and 31.5% in G1, S, and G2/M, respectively), which became more pronounced at higher concentrations. With 20 and 30 µM, the number of parasites in G1 decreased, and the opposite occurred in both S and G2/M phases. In the presence of 20 µM, 41.3% of parasites were in G1, 20% were in S, and 38.7% in G2/M. With 30 µM, 41.7% of epimastigotes were in G1, 21.6% in S and 36.7% in G2/M. After treatment with 40 and 50 µM, the alteration in cell cycle progression was evident, as seen by the decrease of cells in G1 and the increase in S and G2/M. With 40 µM, 24.5% of epimastigotes were in G1, 29.1% were in S and 46.4% in G2/M. With 50 µM, 23.6% of protozoa were in G1, 28.3% in S, and 48.1% in G2/M (Fig. 10A,B and Supplementary Fig. S1 online).

**Figure 11:**
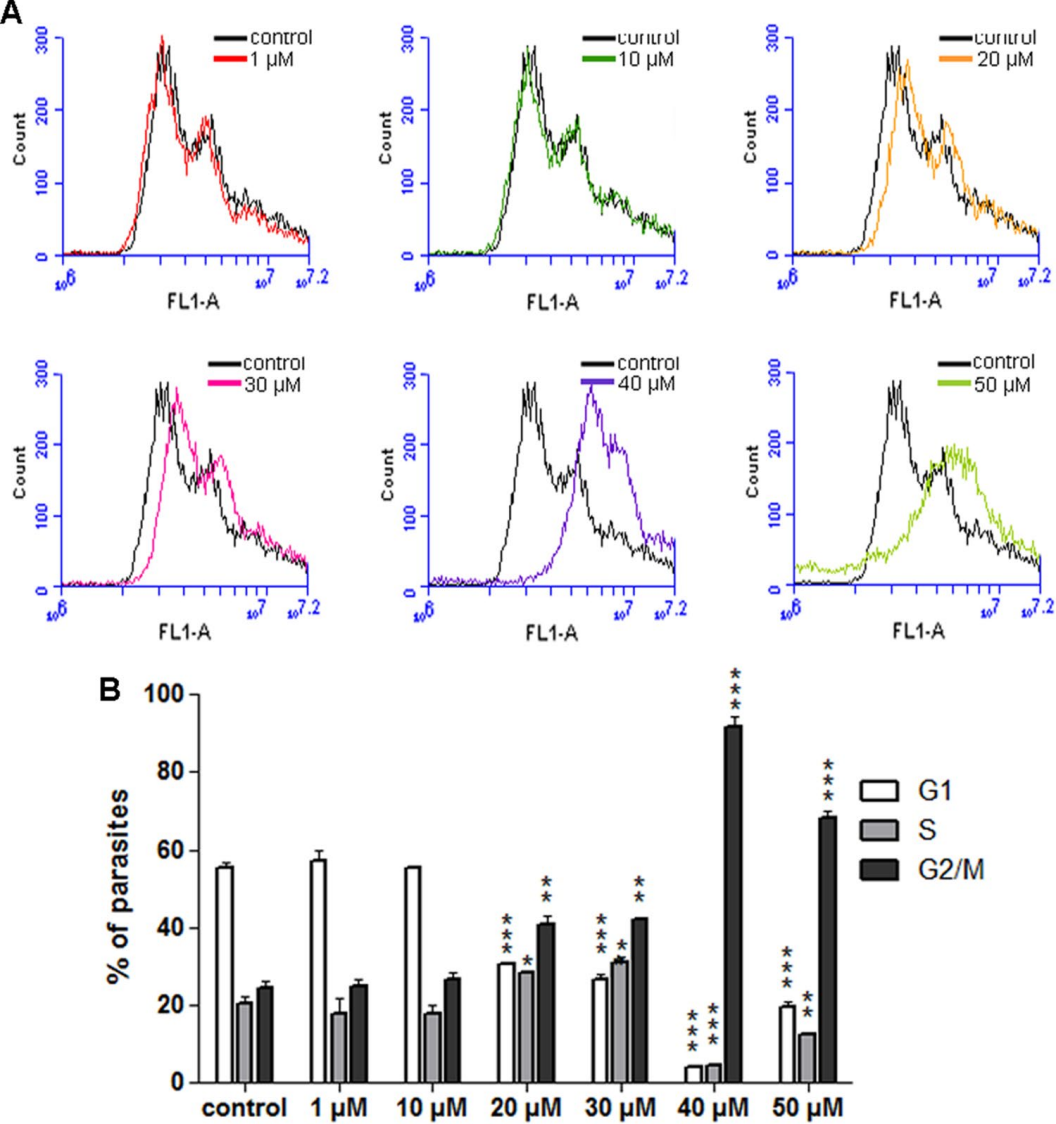
Epimastigotes cell cycle after removal of FEX from the medium. (**A**) Histograms of control and treated parasites. Black and color lines indicate untreated and previously treated epimastigotes, respectively. (**B**) Percentage of parasites in G1, S, and G2/M. The main difference occurred after treatment with 40 and 50 µM when there was a great change in the percentage of parasites in the G1 and G2/M phases. The histograms are representative and the data are the average of two independent experiments (*p > 0.05; **p < 0.05; ***p < 0.001).

For further investigation of the effects of FEX on the *T. cruzi* cell cycle, after 72 h of treatment, the drug was removed from the medium, and the parasites were grown for 3 days. The results of the control group and the parasites previously treated with 1 and 10 µM show that the cell cycle progression was very similar (Fig. 11A,B). In the untreated group, the percentage of epimastigotes in G1 was 55.3%; in S, it was 20.4%; in G2/M, it was 24.3%. With 1 µM, 57.3% of the parasites were in G1, 17.8% in S, and 24.9% in G2/M. With 10 µM, 55.3% of the cells were in G1, 17.9% in S, and 26.8% in G2/M. On the other hand, in the presence of 20 µM, the number of parasites in G1 decreased (30.7%) and increased in G2/M (41%). A similar result was obtained after treatment with 30 µM (26.5% in G1 and 42.3% in G2/M). The greatest differences occurred between 40 and 50 µM. In the presence of 40 µM, the percentage of epimastigotes decreased in G1 and S (to 3.9 and 4.5%, respectively) and increased in G2/M (to 91.6%). With 50 µM, 19.4% of the parasites were in G1, 12.6% were in S, and 68% in G2/M (Fig. 11A,B).

### LLC-MK_2_ viability

**Figure 12:**
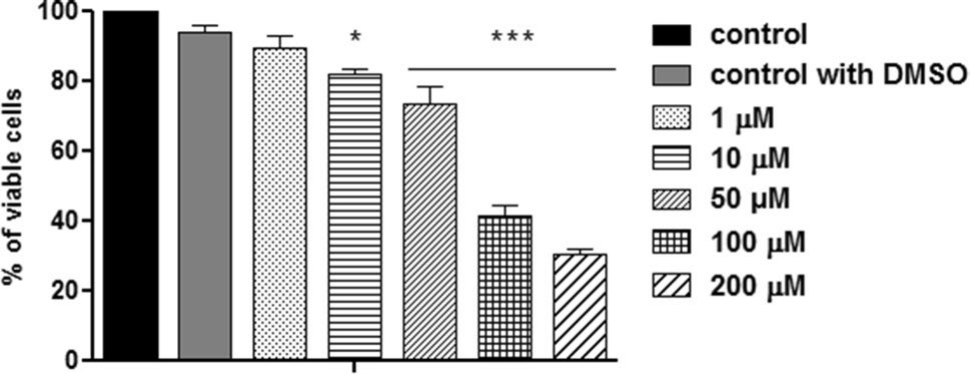
Cell viability of FEX on LLC-MK_2_. FEX caused the reduction of viable cells in a dose-dependent manner, and its CC_50_ value was 80 µM after 72 h of treatment. The data are the average of three independent experiments in triplicate (*p > 0.05; ***p < 0.001).

The cytotoxicity of FEX on LLC-MK_2_ was evaluated after 96 h of treatment to obtain the CC_50_ value (concentration that inhibits the proliferation of host cells by 50%) on mammalian cells and the selectivity index (ratio between cytotoxicity and antiparasitic activity) on *T. cruzi*. FEX caused the reduction of viable cells by approximately 20% at 10 μM and 30% at 50 μM (Fig. 12). In the presence of 100 µM, there was a drastic reduction in the percentage of viable cells (41%). Cell viability was even lower at 200 µM, a condition in which only 30% of the cells were live. Based on these data, the CC_50_ value of FEX was 80 µM ± 10 µM, and its selectivity index was 80 against amastigotes (Table 1).

## Discussion

FEX is a nitroimidazole compound whose biological activity was initially investigated in fungal infections and protozoan parasites. The promising results on *T. brucei* boosted studies that evaluate FEX as a promising compound for antichagasic therapy^1,3,7^. Concerning the mode of action, FEX undergoes oxidative metabolism and forms sulfoxide and sulfone metabolites, which are active against trypanosomatids^3^. Many azoles act on CYP51 (sterol 14α-demethylases), the most conserved P450 cytochrome enzymes involved in sterols biosynthesis. The sequencing of the trypanosomatids genome has shown that the enzymes necessary for this pathway are also present in these organisms. In addition, other reports in the literature show the similarity between the sterol biosynthesis pathway in fungi and trypanosomatids^11–13^.

We show that FEX inhibits both *T. cruzi* epimastigote and amastigote proliferation. The trypanocidal effect against the intracellular parasites was dose-dependent and even more pronounced compared to epimastigotes (Table 1). Furthermore, the 50% cytotoxic concentration on LLC-MK_2_ was 80 µM, which indicates an efficient selectivity index against the pathogenic agent. In 2014, Bahia et al. investigated the cytotoxicity and the antiparasitic activity of FEX on *T. cruzi* Y strain infected macrophages. The authors demonstrated that FEX did not reduce cellular viability when used up to 120 µM and could also decrease the number of infected macrophages in a dose-dependent manner, which was similar to the reference drug^14^.

Although FEX’s in vitro and in vivo trypanocidal properties of are known, its effects on cell organelles, which may constitute potential chemotherapeutic targets, have never been reported by transmission electron microscopy. In this work, we demonstrated that reservosomes had undergone the main ultrastructural changes caused by FEX. Reservosomes are rounded organelles whose matrix presents internal membranes and distinct lipid inclusions found at the posterior end of epimastigotes. They consist of the last organelle of the endocytic pathway in *T. cruzi* epimastigotes. They store lipids, mainly cholesterol esters and ergosterol, and proteins, which will be used as an energy source during parasite metacyclogenesis^15,16^.

We showed an atypical ultrastructure of reservosomes, including many lipid inclusions in its interior, which could be due to the uncommon accumulation of lipids caused by FEX. On the other hand, the intense disorganization of reservosomes, also reported in this work, could be a consequence of endocytosis impairment and could even interfere with epimastigotes proliferation^17–19^. Furthermore, reservosome is the organelle where a high concentration of cruzipain is found^20^, an essential enzyme for parasite virulence, host cell invasion and differentiation, and an important chemotherapeutic target, as well^21^. Adding to the ultrastructural alterations reported in the reservosomes, our results suggest that FEX could also act on cruzipain.

We also demonstrated the presence of many lipid inclusions inside the reservosomes due to treatment with FEX. According to Pereira et al., the cultivation of epimastigotes with a medium supplemented with 50% fetal calf serum caused the formation of lipid inclusions of different shapes in the reservosomes^15^. The accumulation of lipid content leading to the deformation of reservosomes on Y strain epimastigotes was also caused by treatment with lopinavir and nelfinavir, which promote an imbalance in proteolysis and lipid storage, and might inhibit parasite aspartyl peptidases^22^.

Several authors have also reported disorganization of reservosomes due to different drug treatments. Garzoni et al. demonstrated the decrease of electron density and atypical accumulation of lipid inclusions in reservosomes of Y strain epimastigotes after treatment with risedronate^18^. Loss of reservosome morphology was reported after treating epimastigotes (Y strain) with β-lapachone derivatives, whose effects might be related to its basic imidazole moiety^23,24^. In 2007, Adade et al. found swelling, rupture, and loss of content of reservosomes on epimastigotes (Y strain) treated with _L_-Leucine methyl ester. l-Amino acid methyl esters are known for causing lysis of lysosomes of *Leishmania amazonensis* amastigotes probably due to water influx, which leads to organelle welling and disruption^25^. According to Ennes-Vidal et al., treating *T. cruzi* (Y strain) with MDL28170, a calpain inhibitor, led to the disruption of reservosomes with loss of electron density^17^. It is worth mentioning that MDL28170 could act on cruzipain, although it is not a specific inhibitor localized on the reservosomes^17^.

Reservosome injury with endoplasmic reticulum around was also shown after treatment of Y strain epimastigotes with a triazolic naphthofuranquinone by Fernandes et al. This compound acts generating ROS and increasing the number of autophagic vacuoles, suggesting the occurrence of autophagy as a mechanism of action^26^. The treatment of *T. cruzi* epimastigotes (Y strain) with a low concentration of iron complexes initially caused the appearance of spicules inside the reservosomes. At a higher concentration, the authors demonstrated the disappearance of the organelle probably due to degradation. These alterations might have caused an imbalance in endocytosis and affected parasite metabolism^19^.

As we demonstrated, FEX promoted cytoplasmic disorganization and changes in Golgi complex morphology. Similar effects on *T. cruzi* epimastigotes (Y strain) were shown after treatment with two neolignans, licarin A and burchellin^27^, and a quinoxaline derivative^28^. In addition, swelling of Golgi cisternae on epimastigotes and its disruption on amastigotes of *T. cruzi* Y strain were observed after treatment with posaconazole and amiodarone, respectively^29^. Posaconazole is an antifungal drug that targets the sterol biosynthesis pathway and inhibits sterol C14-demethylase, a cytochrome P450-dependent enzyme (CYP51)^30^. Amiodarone, an antiarrhythmic drug, disrupts Ca^2+^ homeostasis and inhibits de novo ergosterol biosynthesis at the level of lanosterol synthesis on *T. cruzi*^31^. Furthermore, the distance among Golgi cisternae on epimastigotes (Y strain) was also an effect of treatment with furan derivatives, as shown by Zuma et al.^32^.

In this study, nuclear heterochromatin underwent unpacking after treatment with a high concentration of FEX and remained in this state even after drug removal from the medium. The less condensed state of chromatin in the presence of FEX might be linked to the blockade of the parasite cell cycle. This effect has been shown on epimastigotes (Y strain) in the presence of topoisomerase inhibitors^33^; chaetocin, a histone methyltransferase inhibitor^34^; 1,10-phenanthroline derivatives^35^; and also when *T. cruzi* was exposed to gamma radiation, which consequently leads to activation of DNA repair mechanisms^36^.

The atypical electron-dense areas next to the kDNA found on trypomastigotes after treatment with FEX resembled what was previously reported on epimastigotes in the presence of Berenil, a DNA minor groove binding drug that inhibits mitochondrial topoisomerase II activity in trypanosomes and disrupts kDNA replication^37^. The drug treatment promoted significant changes in kinetoplast topology, including the appearance of electron-dense regions near the kDNA, which according to the TdT assay, did not contain DNA^38^.

In this work, we have reported similar and distinct ultrastructural alterations among the developmental stages of *T. cruzi* after treatment with FEX. Golgi disorganization was found on epimastigotes and amastigotes. Unpacking nuclear heterochromatin was observed on epimastigotes, but this effect was found neither on amastigotes nor trypomastigotes. It is known that the chromatin on epimastigotes is less compact and is more condensed only in the perinucleolar region and next to the nuclear envelope. On trypomastigotes, the chromatin is more condensed^39^. This difference in the chromatin condensation level could explain epimastigotes' higher susceptibility to the drug. In addition, amastigotes and trypomastigotes presented mitochondrial swelling, which was not evident on epimastigotes during the regular treatment, except in the kinetoplast. The kDNA topology was maintained, but only on trypomastigotes some electron-dense content was found next to the network. Since the kDNA fibers of trypomastigotes are looser compared to epimastigotes and amastigotes, this characteristic could make trypomastigotes more susceptible to the effects of FEX.

## Conclusions

FEX strongly inhibits *T. cruzi* proliferation with an IC_50_ value of 1 µM on amastigotes, and promotes lysis of trypomastigotes. Furthermore, multiple ultrastructural changes in all developmental stages of *T. cruzi* were described, such as the detachment of plasma membrane, disorganization of the reservosomes, disruption of Golgi complex, unpacking of the nuclear heterochromatin and kinetoplast-mitochondrion swelling. Interestingly, fexinidazole might not have a specific cellular target, as different organelles have undergone ultrastructural modifications, which could be compared to the effects of different compounds. However, changes in reservosomes were the most evident. Herein, we first demonstrated FEX’s effects on a trypanosomatid at the ultrastructural level. Taken together, our data might contribute to a better understanding of how FEX works on trypanosomes and demonstrate the main target structures and organelles.

## Material and methods

### Cell culture

*Trypanosoma cruzi* (Y strain) epimastigote forms were grown at 28 °C in liver infusion tryptose (LIT) medium^40^ (Camargo, 1964) supplemented with 10% fetal calf serum (FBS). In addition, LLC-MK_2_ cells (ATCC CCL-7; American Type Culture Collection, Rockville, MD) were cultured in RPMI 1640 medium with Garamycin (GIBCO, Grand Island, NY) 10% FBS at 37 °C in a 5% CO_2_ atmosphere. The amastigotes were maintained in LLC-MK_2_ cells, and the trypomastigotes were obtained from the supernatant of previously infected LLC-MK_2_ cells.

### Trypanocidal effect

FEX was diluted in dimethyl sulfoxide (DMSO) at 5 mM and 20 mM. To investigate the effect on *T. cruzi* epimastigote proliferation, 1 × 10^6^ cells/ml were incubated in LIT supplemented with 10% FBS. After 24 h of initial growth, 1, 10, 20, 30, 40, and 50 μM were added to the culture. Every 24 h for up to 96 h, parasites were counted using BD Accuri C6 flow cytometer (BD Biosciences, USA), and the data were analyzed using BD Accuri C6 software. To evaluate the reversibility of FEX on epimastigotes proliferation, the concentrations mentioned above were added to the medium after 24 h of growth. After 72 h of treatment, cultures were washed with LIT to remove the drug from the medium. Then, parasites were incubated with fresh medium supplemented with FBS for another 72 h. Parasite counting and data analyses were performed as described above.

To evaluate the effect against trypomastigotes, 1 × 10^4^ parasites/ml from the supernatant of infected LLC-MK_2_ cells were collected and incubated in RPMI with 10, 30, 50, and 70 μM for 24 h. After this period, treated and non-treated parasites were counted by flow cytometry, as described above, taking into account the forward and side scatter patterns of the control group.

To evaluate the effect on intracellular amastigotes, LLC-MK_2_ (5 × 10^4^) and trypomastigotes (250 × 10^4^) were plated in a 96-well plate, and after 24 h of interaction, 0.5, 1, 5, and 10 µM were added. After 72 h of treatment, cells were fixed in 4% freshly prepared formaldehyde diluted in PBS (pH 7.2) for 10 min at room temperature and were washed in distilled water. The cells were stained with 2 μg/mL Hoechst for 1 h, protected from light at room temperature, and then washed twice in distilled water. After that, the number of cells, infected cells, and intracellular parasites were obtained using a high throughput screening platform (ImageXpress Micro—Molecular Devices). Three independent experiments in triplicate were performed. The IC_50_ (concentration that inhibits 50% of the proliferation of the replicative form) and LD_50_ (concentration that causes lysis in 50% of the trypomastigotes) values were calculated by fitting the values to a non-linear curve analysis. The regression analyses were performed with SigmaPlot 10 software.

### Cell viability

To evaluate FEX toxicity, LLC-MK_2_ cells were plated in a 96-well plate, using 2.5 × 10^5^ cells/well, and after 24 h of growth, different concentrations (1; 10; 50; 100 and 200 μM) of FEX were added in RPMI medium. Regarding parasite viability, *T. cruzi* epimastigotes (1 × 10^6^ cells/ml) were incubated in LIT supplemented with 10% FBS, and after 24 h of initial growth, 1, 10, 20, 30, 40, and 50 μM FEX was added to the medium. Cell viability was measured after 96 h of treatment by CellTiter 96^®^ Aqueous MTS Assay (Promega, United States)^41^. MTS/PMS assay reaction was quantified by optical density measurement at 490 nm using a SpectraMax M2/M2 spectrofluorometer (Molecular Devices, United States). Three independent experiments in triplicate were performed. The CC_50_ values were calculated by fitting the values to a non-linear curve analysis. The regression analyses were performed with SigmaPlot 10 software.

### Scanning electron microscopy

Scanning electron microscopy was used to evaluate alterations on *T. cruzi* morphology and surface. After treatment with FEX, parasites were fixed as described above and adhered to poly-l-lysine coated microscope coverslips for 10 min. The samples were post-fixed as previously described for 30 min, dehydrated (with ethanol), critical point dried in CO_2,_ and ion sputtered. The samples were observed under a Quanta 250 scanning electron microscope (SEM; FEI Company, The Netherlands). To evaluate the reversibility of FEX on epimastigotes morphology, the same procedure as in item 2.4 was carried out.

### Transmission electron microscopy

Transmission electron microscopy was used to investigate the modifications caused by FEX on organelle ultrastructure. Thus, treated and non-treated parasites were fixed in 2.5% glutaraldehyde diluted in 0.1 M cacodylate buffer (pH 7.2) for 1 h at room temperature and were washed in the same buffer. Cells were post-fixed in 1% OsO_4_ and 0.8% potassium ferricyanide for 1 h, protected from light at room temperature. The samples were washed three times in the same buffer, dehydrated in a graded series of acetone, and embedded in Epon (Electron Microscopy Sciences, Hatfield, PA). Ultrathin sections were stained with uranyl acetate for 45 min and lead citrate for 5 min. Then, the samples were observed using a Tecnai™ Spirit TEM transmission electron microscope (Zeiss, Oberkochen, Germany). To evaluate the reversible effect of FEX on epimastigotes ultrastructure, FEX was added to the medium after 24 h of growth. After 72 h of treatment, cultures were washed with LIT to remove the drug from the medium. Then, parasites were incubated with fresh medium supplemented with FBS for another 72 h and were prepared as described above.

### Cell cycle analyses by flow cytometry

To analyze the progression of the *T. cruzi* cell cycle during treatment with FEX, parasites were fixed in 0.25% formaldehyde in PBS (pH 7.2) for 5 min and then washed and resuspended in 70% cold ethanol for 30 min. Subsequently, cells were washed and incubated with 5 µM SYTOX^®^ Green (Invitrogen, USA) for 30 min at room temperature. The analysis was performed on a BD Accuri C6 flow cytometer (BD Biosciences, USA), and the BD Accuri C6 software analyzed the data. Two independent experiments performed this analysis.

To evaluate the reversible effect of FEX on the epimastigotes cell cycle, the drug was added to the medium after 24 h of growth. After 72 h of treatment, cultures were washed with LIT to remove the drug from the medium. Then, parasites were incubated with fresh medium supplemented with FBS for another 72 h. The analyses were performed as described above.

### Fluorimetric analysis using Nile Red

To quantify the number of neutral lipids after treatment with FEX, epimastigotes were washed twice in PBS (pH 7.2). They were incubated in 10 µg/ml Nile Red for 20 min at room temperature, protected from light. Then, parasites were washed in PBS and transferred to a black 96-well microplate. Nile Red fluorescence was determined in SpectraMax M2/M2 espectrofluorometer (Molecular Devices, United States) with excitation at 485 nm and emission at 535 nm. Three independent experiments in triplicate were performed.

### Statistical analyses

The statistics were assessed using the one-way or two-way analysis of variance (ANOVA) test followed by Bonferroni's multiple-comparison test in the GraphPad Prisma 5 software. Results were considered statistically significant when P was < 0.05(*), < 0.01(**), and < 0.001(***).

## Supplementary Information

Supplementary Figure S1.

## Data Availability

All electron microscopy data created during this research is available at https://www.ebi.ac.uk/biostudies (TMP_1663108258007). All cytometry data created during this research is available at http://flowrepository.org/ (FR-FCM-Z5PE).
