# Supplementary material for: Fexinidazole interferes with the growth and structural organization of Trypanosoma cruzi
Source: Sci Rep. 2022 Nov 27;12:20388. doi: 10.1038/s41598-022-23941-z (PMC9701812; doi:10.1038/s41598-022-23941-z)
Supplement: Supplementary file 1 — Supplementary Figure S1. [file 41598_2022_23941_MOESM1_ESM.pdf]

Fexinidazole interferes with the growth and structural organization of *Trypanosoma cruzi*

Aline Araujo Zuma[1*] and Wanderley de Souza[1,2]

[1]Laboratorio de Ultraestrutura Celular Hertha Meyer, Instituto de Biofísica Carlos Chagas Filho, Universidade Federal do Rio de Janeiro; Av. Carlos Chagas Filho, 373, Centro de Ciências da Saúde, Cidade Universitária, Ilha do Fundão, 21491-590, Rio de Janeiro, RJ, Brazil; Phone number: +55 21 39386580

[2] Centro Multidisciplinar de Pesquisas Biológica-CMABio, Escola Superior de Ciências da Saúde, Universidade do Estado do Amazonas-UEA, Av. Carvalho Leal, 1777 - Cachoeirinha, CEP 69065-000, Manaus-AM

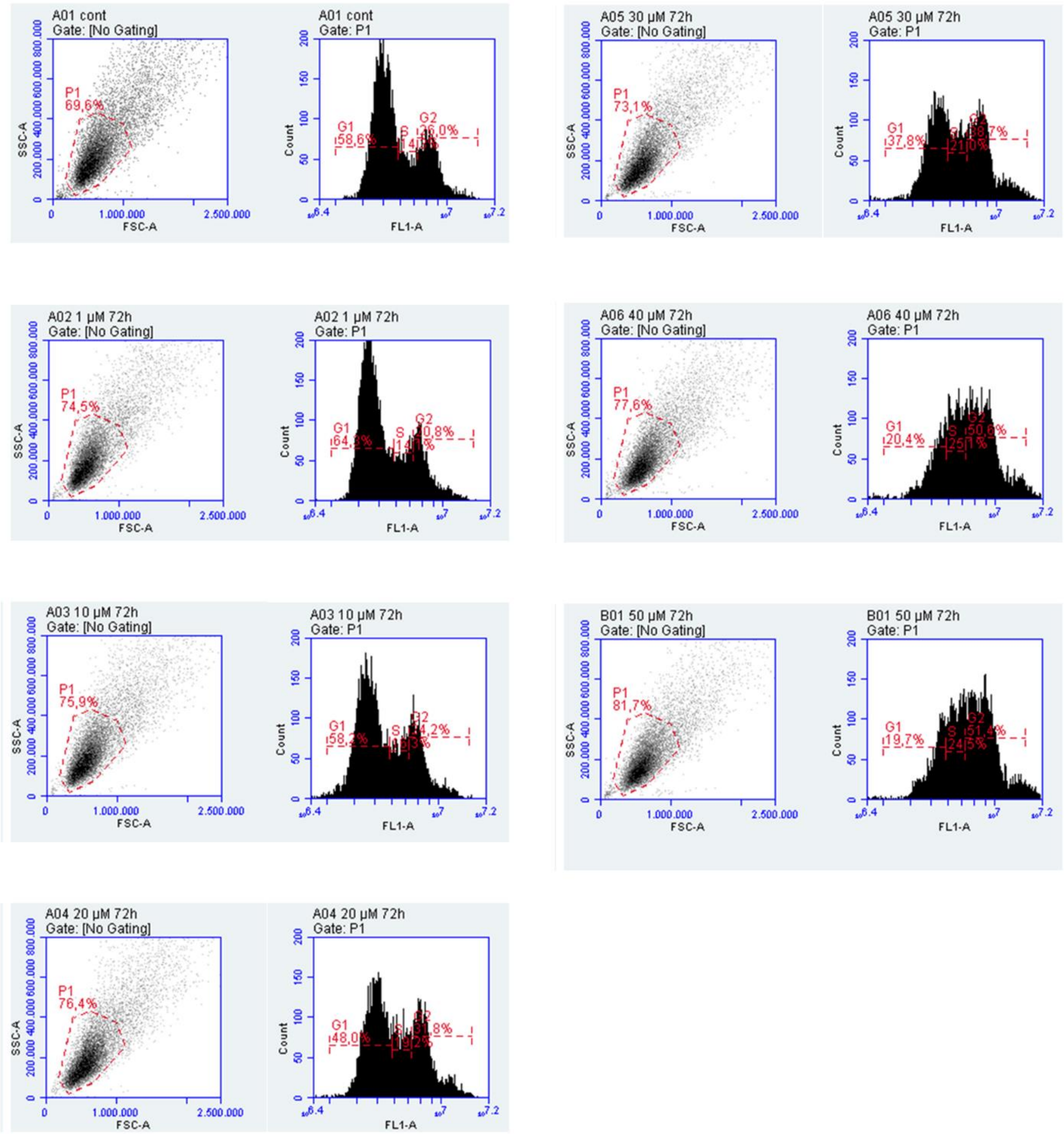

Supplementary Fig. S1: Epimastigotes cell cycle in the presence of FEX for 72 hours. The regions on the histogram that corresponds to each cell cycle phase are highlighted and treated parasites were compared to the control group. The percentage of cells in each phase of the cell cycle was obtained using BD Accuri C6 software.
